# Cohort profile: congenital Zika virus infection and child neurodevelopmental outcomes in the ZEN cohort study in Colombia

**DOI:** 10.4178/epih.e2020060

**Published:** 2020-08-31

**Authors:** Maritza Gonzalez, Van T. Tong, Helena Rodriguez, Diana Valencia, Jacqueline Acosta, Margaret A. Honein, Martha L. Ospina, The ZEN Study Team

**Affiliations:** 1 Instituto Nacional de Salud (INS), Bogotá, Colombia; 2 Centers for Disease Control and Prevention (CDC), Atlanta, GA, USA; 3 Vysnova Partners, Inc., Landover, MD, USA

**Keywords:** Zika virus, Congenital abnormalities, Closed cohort studies, Pregnancy, Infant, Infant development

## Abstract

*Zika en Embarazadas y Niños* (ZEN) is a prospective cohort study designed to identify risk factors and modifiers for Zika virus (ZIKV) infection in pregnant women, partners, and infants, as well as to assess the risk for adverse maternal, fetal, infant, and childhood outcomes of ZIKV and other congenital infections. ZIKV infection during pregnancy may be associated with long-term sequelae. In the ZEN cohort, 1,519 pregnant women and 287 partners were enrolled from 3 departments within Colombia between February 2017 and January 2018, as well as 1,108 infants born to the pregnant women who were followed to 6 months. The data include baseline questionnaires at enrollment; repeated symptoms and study follow-up questionnaires; the results of lab tests to detect ZIKV and other congenital infections; medical record abstractions; infant physical, eye, and hearing exams; and developmental screening tests. Follow-up of 850 mother-child dyads occurred at 9 months, 12 months, and 18 months with developmental screenings, physical exams, and parent questionnaires. The data will be pooled with those from other prospective cohort studies for an individual participant data meta-analysis of ZIKV infection during pregnancy to characterize pregnancy outcomes and sequelae in children.

## INTRODUCTION

Zika virus (ZIKV) is transmitted primarily through the bite of an infected *Aedes* species mosquito; however, it can also be transmitted between sexual partners or from mother-to-child in utero or during delivery [1,2]. Numerous studies have documented the serious consequences of ZIKV infection during pregnancy, including fetal or infant brain and eye abnormalities, such as intracranial calcifications and microcephaly [3-7]. Key knowledge gaps still exist, such as understanding the full spectrum of adverse outcomes in pregnant women, fetuses, and infants associated with ZIKV infection in pregnancy; the relative contributions of sexual transmission and mosquito-borne transmission to the occurrence of infections in pregnancy; and the risk of adverse fetal and infant outcomes by gestational week of maternal infection or presence of ZIKV symptoms. Limited evidence suggests that ZIKV infection during pregnancy may be associated with long-term sequelae, including poor neurodevelopmental outcomes for those without congenital abnormalities [8-13]; however, the lack of an adequate comparison group in existing analyses limits the interpretation of whether these adverse outcomes can be attributed to ZIKV infection. There is a need to describe the risk of adverse developmental outcomes in infants and children following ZIKV exposure in utero.

The Colombian *Instituto Nacional de Salud* (INS) in collaboration with the United States Centers for Disease Control and Prevention (CDC) established the *Zika en Embarazadas y Niños* (ZEN) prospective cohort study in multiple Colombian cities. The primary objectives were to: (1) identify risk factors for ZIKV infection in pregnant women, partners, and infants; (2) assess the risk for adverse maternal, fetal, infant, and childhood outcomes associated with ZIKV infection and other congenital infections; and (3) assess modifiers of risk.

## STUDY PARTICIPANTS

**Figure 1.**
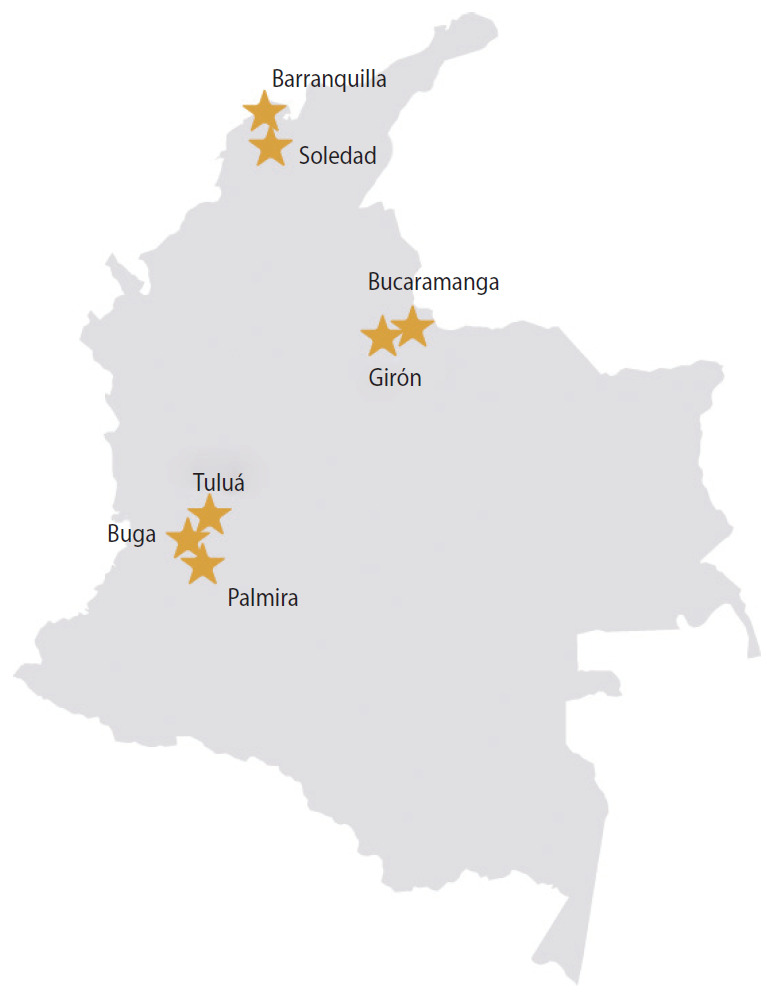
*Zika en Embarazadas y Niños* (ZEN) cohort study sites in Colombia (2017-2020). A total of 13 prenatal care clinics participated in study enrollment in 3 departments of Colombia. The Atlántico study site included 2 clinics in Barranquilla and 2 in Soledad. The Santander study site included 3 clinics in Bucaramanga and 1 in Girón. The Valle del Cauca study site included 2 clinics in Buga, 2 in Tuluá, and 1 in Palmira.

Pregnant women aged 16 years or older were recruited in their first trimester of pregnancy from participating public and private prenatal care clinics in Colombia. The 13 participating clinics were located in cities in 3 departments: Atlántico, Santander, and Valle del Cauca (Figure 1), representing 50,000 live births in 2017 [14]. Cities/clinics were selected based on national surveillance data indicating ZIKV disease in the prior year, a large volume of pregnant women receiving prenatal care, and support from local health authorities.

**Figure 2.**
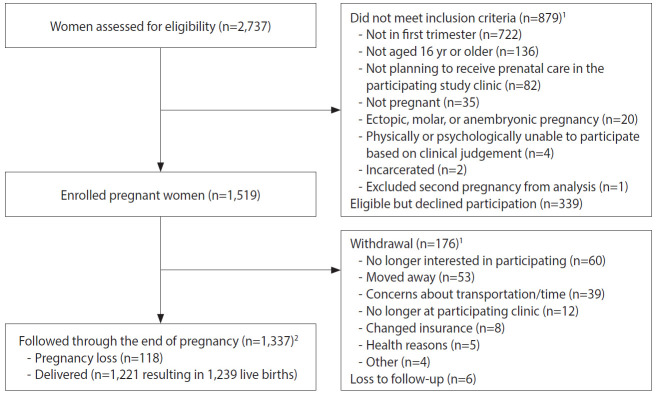
Flow-chart of enrollment and follow-up for pregnant women, *Zika en Embarazadas y Niños* (ZEN) cohort study (2017-2020). ^1^Categories are not mutually exclusive. ^2^Includes two multiple gestation pregnancies, which resulted in discordant pregnancy outcomes.

Pregnant women were recruited by study staff in clinic waiting rooms, by referral from prenatal care providers, or by self-referral after seeing recruitment brochures or posters. Among the pregnant women screened for eligibility (n=2,737), 879 (32.1%) were excluded because they did not meet eligibility criteria (Table 1 and Figure 2).

Some eligible women (n=339, 12.4%) declined participation after screening. One participant was enrolled twice, and the second pregnancy was excluded from the analysis. A total of 1,519 pregnant women were enrolled across sites from February 2017 to January 2018. Upon enrollment, pregnant women received ZIKV prevention kits with educational materials, condoms, diethyltoluamide (DEET)-containing mosquito repellent, an appointment card, and a thermometer.

**Figure 3.**
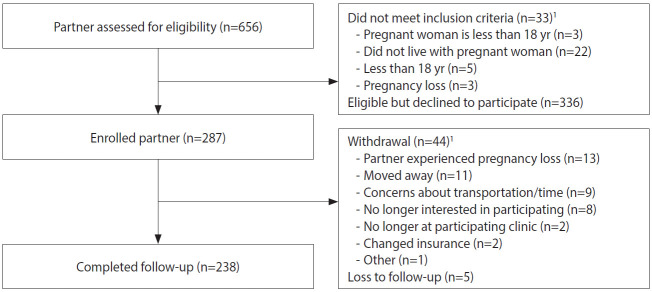
Flow-chart of enrollment and follow-up for male partners, *Zika en Embarazadas y Niños* (ZEN) cohort study (2017-2020). ^1^Categories are not mutually exclusive.

For enrolled pregnant women ≥ 18 years, staff asked if they had a male partner who lived with them, and if staff could contact him for participation. Of the 1,399 enrolled pregnant women aged ≥ 18 years, 156 (11.1%) did not have a male partner or one who lived with them, 460 (32.9%) did not agree to have their partners contacted, 4 did not respond to the question, and 779 (55.7%) agreed for staff to contact their partner. Of those who agreed, 123 partners could not be reached. Among the 656 men screened, 33 (5.0%) were not eligible (Table 1 and Figure 3), and 336 (51.2%) declined participation. A total of 287 male partners were enrolled across sites from February 2017 to February 2018.

**Figure 4.**
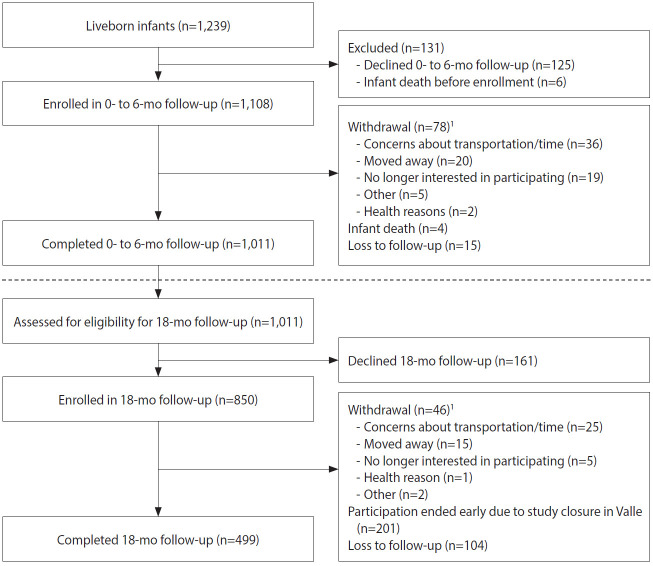
Flow-chart of enrollment and follow-up for infants in the 0- to 6-month follow-up and children in the 18-month follow-up, *Zika en Embarazadas y Niños* (ZEN) cohort study (2017-2020). ^1^Categories are not mutually exclusive.

Among the 1,519 enrolled pregnant women, 1,337 (88.0%) participated throughout their pregnancies, with 1,221 women delivering at least 1 liveborn infant (n=1,239 infants; Figure 2). A total of 1,108 infants were enrolled in the first phase of follow-up, which occurred from birth through approximately 6 months of age (Figure 4). Around the 6-month follow-up, parents (or guardians) were assessed for interest and eligibility for themselves and their infant to participate in the second phase of follow-up through 18 months of age. Parents were given a study kit at enrollment and each follow-up, which included developmentally appropriate toys and supplies and health education materials. Of the 1,011 infants who completed the 6-month follow-up, all met eligibility criteria for the 18-month follow-up, but the parents of 161 (15.9%) infants declined further participation (Figure 4). A total of 850 infants (including 11 multiple birth sets) and their corresponding 839 parents/legal guardians enrolled in the 18-month follow-up. A total of 499 infant-parent pairs completed follow-up to 18 months.

### Participant follow-up

Pregnant women were administered baseline and symptoms questionnaires at enrollment, and a venous blood sample was collected for ZIKV, dengue virus (DENV), chikungunya virus (CHIKV), and syphilis, toxoplasmosis, rubella, cytomegalovirus, and herpes I and II (STORCH) testing (Supplementary Material 1). Women were followed every 2 weeks until the end of pregnancy, alternating between monthly study clinic and interval visits (about 2 weeks after the monthly study clinic visits). During the study clinic visits, study follow-up and symptoms questionnaires were administered, and a blood sample was collected. During the interval visits, a symptoms questionnaire was administered, and a urine sample was collected. If at any time a pregnant woman reported experiencing two or more ZIKV-compatible symptoms within the previous week, study staff asked for a blood sample and administered a symptoms questionnaire. If a woman tested positive for ZIKV by real-time reverse transcription polymerase chain reaction (rRT-PCR) at any time during the study, she was asked to provide a blood sample every 2 weeks, in lieu of urine collection, until 2 consecutive samples tested negative for ZIKV by rRT-PCR. At delivery or within the first 10 days postpartum, study staff administered a follow-up and symptoms questionnaire and collected blood from women to test for STORCH, ZIKV, DENV and CHIKV. For pregnancy losses, fetal or placental tissues were obtained (collected by the clinician), maternal venous blood was collected, and a symptoms questionnaire was administered.

At enrollment, male partners were administered a baseline and symptoms questionnaire, and a venous blood sample was collected (Supplementary Material 2). Through the end of his partner’s second trimester of pregnancy, monthly urine samples and symptoms questionnaires were collected to monitor for incident ZIKV infection. If the male partner reported ZIKV-compatible symptoms, study staff asked for a blood sample and administered a symptoms questionnaire. Men who tested positive for ZIKV were asked to provide semen samples every 2 weeks, in lieu of urine collection, until 2 consecutive negative ZIKV tests or the end of his partner’s pregnancy.

Infants had a venous blood sample collected at delivery or within 10 days postpartum to test for in utero ZIKV infection, and a symptoms questionnaire was administered to the parent about infant ZIKV symptoms and healthcare visits (Supplementary Material 3). Infants enrolled in the 6-month follow-up had urine samples collected using an adhesive pediatric urine bag, and infant symptoms questionnaires were administered every 2 weeks. Standard anthropometric measurements, neurodevelopmental evaluations, and hearing and vision screening were also conducted.

For parent-child pairs enrolled in the 18-month follow-up, standard anthropometric measurements were taken, and neurodevelopmental evaluations were administered to the child at 6 months, 9 months, 12 months, and 18 months (Supplementary Material 4). Parents were administered questionnaires to understand their health and family environment. At 12 months, children also received standard eye examinations. Follow-up at the Valle de Cauca site (5 clinics) was ended early in March 2019; complete 18-month study appointments for parent-child pairs were exclusive to the remaining 8 clinic sites in Atlántico and Santander.

### Ethics statement

The study protocol was approved by the INS Ethics and Methods Committee and the CDC Institutional Review Board. All participants provided informed consent. Participation was voluntary, and they could choose to opt out of study activities or withdraw at any time.

## MEASUREMENTS

### Questionnaires

The baseline enrollment questionnaire for pregnant women included information about demographics; behavioral, sexual, environmental, and occupational exposures; medical and reproductive history; knowledge and perceptions of ZIKV; previous history of and risk factors for ZIKV infection; recent symptoms of ZIKV infection among household members; and history of DENV, CHIKV, and yellow fever infections and yellow fever vaccination. The monthly follow-up questionnaire for pregnant women focused on assessing changes in selected exposures or risk factors throughout the pregnancy. Male partners completed a similar baseline questionnaire at enrollment; no follow-up questionnaires were completed by male partners. The adult symptoms questionnaire included questions about ZIKV-compatible symptoms (e.g., fever, rash, red eyes, joint pain or swelling) since the last study visit, other symptoms that could be associated with ZIKV infection, date of symptom onset, and symptom duration. In addition to these questions, the infant symptoms questionnaire also included questions about feeding, crying behaviors and health care encounters since the last study visit.

For parent-child pairs participating in the 18-month follow-up, enrollment and follow-up questionnaires were administered to parents to assess household characteristics (e.g., number and age of children); caregiving responsibilities and family support; and other household environmental risk factors potentially impacting child neurodevelopment (e.g., pesticide use, smoking, and illicit drug use). Two questionnaires were administered to understand parents’ mental health and the family environment. The Parenting Stress Index-Short Form (PSI-SF) assesses the sources and types of stress that a parent can experience [15]. The Center for Epidemiologic Studies Depression Scale 10 (CES-D 10) is a self-reported measure of depression [16]; parents who received a score equal to or above 10 were considered at risk for depression and were referred to a clinician for further evaluation.

### Laboratory testing

The Trioplex rRT-PCR assay (provided by CDC under an emergency use authorization) was used to simultaneously detect RNA from ZIKV, DENV, and CHIKV in serum, urine, semen, fetal tissue, and other specimen types [17]. The ZIKV Detect Immunoglobulin M (IgM) Antibody Capture ELISA 1.0 and 2.0 (InBios International Inc., Seattle, WA, USA) was used to detect anti-Zika IgM antibodies in maternal serum at enrollment and delivery, and in infant serum collected within 10 days after delivery. AntiDENV antibodies were detected using the Panbio Dengue IgM Capture ELISA (Panbio/Abbott Laboratories, Abbott Park, IL, USA) to rule out cross-reactivity against DENV, due to high antigenic homology between DENV and ZIKV. Maternal serum samples at enrollment and delivery were tested for IgG and IgM antibodies for STORCH pathogens. An algorithm was developed for priority testing, in which IgG antibodies were first tested, followed by IgM antibodies if positive.

Biological specimens were held at 2-6°C before storage at -20°C. The rRT-PCR testing was performed by the INS arbovirus laboratory in Bogotá and public health laboratories in Barranquilla and Cali. The ZIKV and DENV IgM assays were conducted at the INS reference laboratory. STORCH testing was conducted by commercial reference laboratories in Colombia. Remaining specimens were stored at the INS laboratory at -70°C.

### Child physical assessments

Physical assessment, including anthropometric measurements, were conducted according to a schedule (Supplementary Materials 3 and 4). Cranial ultrasonography to detect brain abnormalities was performed once between delivery and 6 months of age. Standard hearing screenings were conducted at 1 month, 3 months, and 6 months of age. Routine eye examinations were conducted between 1 month and 3 months of age, at 6 months of age, and at 12 months of age. Retinal eye exams were conducted once during the first 6 months of age for infants with confirmed or possible ZIKV infection, infants with an abnormal routine eye exam, and for a sample of unexposed infants.

### Child developmental evaluations

Four developmental screening/evaluation tools available in the Spanish language were conducted per the study schedule by trained study staff (Supplementary Materials 3 and 4). The Ages and Stages Questionnaires, Third Edition (ASQ-3) is a validated parent-report screening tool used to monitor children’s development across the primary developmental domains [18]. The *Escala Abreviada de Desarrollo*, Third Edition (EAD-3) is a clinician-administered screening tool used by the Colombian Ministry of Health to monitor children’s development across the primary developmental domains (with the exception of the cognitive domain); the EAD-3 has been validated, with norms developed, within the Colombian population [19]. The Ages and Stages Questionnaires: Social-Emotional, Second Edition (ASQ:SE-2) is a validated parent-report screening tool that is used to identify possible challenges related to children’s social and emotional development [20]. The Bayley Scales of Infant and Toddler Development, Third Edition (BSID-III) is a validated clinician-administered evaluation tool that examines cognitive, language, and motor development to identify children with possible developmental delays [21]. Children with scores on any developmental tool suggesting concerns about their developmental status were referred to a clinician.

### Medical record abstraction

Maternal prenatal care records were abstracted for information on obstetric history, pre-existing conditions, prenatal care, medication use, ultrasound examinations, and hospitalization during pregnancy. Delivery records were abstracted for information on delivery outcome, maternal complications, and maternal death. Infant birth records were abstracted for information on gestational age at delivery, physical appearance and measurements at birth, infant complications at birth, imaging or laboratory test results, neonatal intensive care unit admission, infant death, and medical diagnoses made up to 10 days after birth. No abstraction was performed for children following 10 days after birth.

### Data management and analysis

Data were entered into REDCap version 7 (Research Electronic Data Capture, Nashville, TN, USA) and analyzed using SAS version 9.5 (SAS Institute Inc., Cary, NC, USA).

## KEY FINDINGS

Data are presented for the pregnancy and male partner cohort. Among the 1,519 enrolled pregnant women, the median gestational age at enrollment was 10 weeks (Table 2).

The largest proportions of enrolled pregnant women were 18-24 years old (44.3%), had at least secondary education (48.1%), had public health insurance (68.5%), were living with a partner (73.7%), and were from Atlántico (41.1%). Of 1,519 enrolled pregnant women, 176 (11.6%) withdrew and 6 (0.4%) were lost to follow-up at time of delivery (Figure 2). Of the remaining 1,337 women who remained in the study, 118 (8.8%) had a pregnancy loss and 1,221 (83.8%) had a live birth (includes two multiple gestation pregnancies, which resulted in discordant pregnancy outcomes). All 1,519 enrolled pregnant women had at least 1 serum sample obtained for ZIKV testing; 93% had at least 1 urine sample, and 2 had amniotic fluid samples (taken for other clinical indications); placental tissue samples from 4 losses were submitted for ZIKV testing. On average, pregnant women had 5 serum samples (range, 1 to 11) and 5 urine samples (range, 0 to 15) collected. The total number of samples collected per pregnant woman for ZIKV testing ranged from 1 to 18, with a mean of 9. Sixteen women (1%) had at least 1 positive sample for ZIKV by rRT-PCR.

Among the 287 male partners, the largest proportions of men were 25-34 years of age (49.1%), had at least secondary education (48.1%), had private health insurance (47.1%), and were from Atlántico (41.5%) (Table 2). All male partners had a serum sample taken at enrollment, and 91% had at least 1 urine sample taken for ZIKV testing during their partners’ pregnancy. One participant had semen samples collected. On average, male partners had 3 urine samples (range, 0 to 6) collected for ZIKV testing. The total number of samples collected per male partner ranged from 1-7, with a mean of 4. Two men (0.7%) had at least 1 positive sample for ZIKV by rRT-PCR. Compared to pregnant women with no male partner enrolled, women with a male partner enrolled had higher educational achievement, were more likely to have private insurance, and were more likely to be married (Supplementary Material 5).

## STRENGTHS AND LIMITATIONS

The ZEN cohort study aims to provide a prospective evaluation of adverse maternal, fetal, infant, and early childhood outcomes associated with ZIKV and other infections during pregnancy. The study results may guide recommendations for preventing congenital infections including ZIKV, improve counseling of patients about the risks of congenital infections, and help agencies prepare to monitor neurodevelopment and provide services to affected children and their families.

This cohort has several strengths. First, the study design allowed for intensive and frequent follow-up to examine incident infections during pregnancy and infancy. Second, this is one of the few ZIKV cohort studies that included male partners, which will provide information on the protective and risk behaviors for male-to-female sexual transmission of ZIKV in pregnancy. Third, the study collected information on multiple prenatal exposures and risk factors for poor pregnancy and child outcomes that can be examined along with physical and developmental outcomes at several time points between delivery and 18 months of age. In addition, data from the ZEN cohort can help fill gaps in knowledge about the developmental trajectory of children in Colombia more generally, and can also be used as a comparison population for other Colombian cohorts of children exposed in utero to ZIKV.

The ZIKV outbreak was unprecedented in its devastating impact on pregnant women; however, the outbreak had waned by early 2017 when enrollment in the ZEN cohort began [22]. The ZIKV infection rate in this cohort was substantially lower (1%) than the 10% incidence at the peak of the epidemic; thus, the study is under-powered to study the association between ZIKV and adverse pregnancy outcomes. A prospective cohort study enrolling high-risk pregnancies found that 8% of participants at any gestational age were ZIKV-positive by rRT-PCR, and that Zika infection was associated with an increased risk of microcephaly, but not of some adverse pregnancy outcomes (e.g., preterm delivery, small for gestational age) [23,24]. Other studies enrolling pregnant women during the first trimester to monitor incident infections have yet to publish their results [25-27]. Data from the ZEN cohort will be pooled with those from other prospective cohort studies for an individual participant data meta-analysis of ZIKV infection during pregnancy to characterize pregnancy outcomes and longer-term sequelae in children [28]. A third of pregnant women did not consent for their partners to be contacted and only half of the eligible partners agreed to participate in the study, which aligned with the prenatal care appointments when possible. Women with a male partner enrolled had higher educational achievement, were more likely to have private insurance, and were more likely to be married. Furthermore, the scope of the research study was broadened to include additional congenital infections (e.g., cytomegalovirus, lymphocytic choriomeningitis virus), which will allow us to answer key questions about the prevalence and impact of these infections on pregnancy and child neurodevelopment.

There are several lessons learned that could be applied in future infectious disease outbreaks in which pregnant women are especially vulnerable. The study leveraged the existing strong national surveillance and laboratory testing infrastructure of Colombia; however, challenges existed in assembling a cohort in the midst of an epidemic. Delays in enrollment were in part due to the need to build local capacity, consolidate research support, and obtain the required reviews in the United States and Colombia [29]. Study investigators worked collaboratively with local health authorities to identify sites and qualified study staff, develop culturally appropriate materials, and conduct training to expand ZIKV testing capacity and to conduct developmental assessments. The ZEN study established a research network in Colombia that could be leveraged for studies of pregnant women and infants. Finally, the capacity-building of healthcare workers and the emphasis on recommended developmental evaluation for all children, including those affected by ZIKV and other congenital infections, strengthened the health systems in Colombia to improve the health of women, children, and families.

## DATA ACCESSIBILITY

Opportunities for collaboration should be directed to the coinvestigator, Diana Valencia (ile9@cdc.gov).

## Figures and Tables

**Table 1.** Inclusion and exclusion criteria for pregnant women, their male partners, and their children in the 18-month follow-up, *Zika en Embarazadas y Niños* (ZEN) cohort study (2017-2020)

Pregnant women	Male partners	Subset of parent/children in the 18 mo follow-up
Inclusion criteria		
Aged 16 yr or older	Aged 18 yr or older	Parent or individual is the legal guardian of the enrolling child
Speaks Spanish	Female partner is aged 18 yr or older	Parent speaks Spanish
Confirmed pregnancy (blood beta human chorionic gonadotropin [hCG], urine beta-hCG, or ultrasound)	Speaks Spanish	Parent lives with the child
Gestational age ≤14 wk 6 d at time of consent	Lives in the same household as female partner already enrolled in the study (married or domestic partner)	Parent anticipates living with the child for the duration of the study
Attending or willing to attend prenatal care visits at collaborating clinics	Female partner is enrolled in ZEN and agrees that the male partner can be asked to participate	Child is <12 mo (1 yr) of age at the time of enrollment
Exclusion criteria		
Incarcerated	Incarcerated	Parent is unable or unwilling to consent to proposed study activities or give permission for child to engage in proposed study activities
Unable or unwilling to consent to study activities	Unable or unwilling to consent to study activities	
Physically or psychologically unable to participate, based on judgement of study staff or clinician	Physically or psychologically unable to participate, based on judgement of study staff or clinician	Parent is not physically or psychologically able to participate based on clinical judgement

**Table 2.** Demographics of pregnant women and their male partners at enrollment, *Zika en Embarazadas y Niños* (ZEN) cohort study (2017-2020)

Variables	Pregnant women (n=1,519)	Male partners (n=287)
Median gestational week of pregnant woman at enrollment of participant	10 (IQR: 7, 12)	9 (IQR: 7, 12)
Age (yr)		
16-17	120 (7.9)	NA
18-24	673 (44.3)	83 (28.9)
25-34	610 (40.2)	141 (49.1)
≥35	116 (7.6)	63 (22.0)
Highest level of education		
Primary or less	246 (16.2)	47 (16.4)
Secondary	730 (48.1)	138 (48.1)
Technical or university	540 (35.5)	102 (35.5)
Missing	3 (0.2)	0 (0.0)
Type of health insurance		
Private	449 (29.6)	135 (47.0)^1^
Public	1,041 (68.5)	131 (45.6)^1^
Not insured	21 (1.4)	11 (3.8)^1^
Missing	8 (0.5)	10 (3.5)^1^
Relationship status		
Married	195 (12.8)	NA
Living with partner	1,119 (73.7)	NA
Single, divorced, widowed, and other	201 (13.2)	NA
Missing	4 (0.3)	NA
Study site^2^		
Atlántico	625 (41.1)	119 (41.5)^1^
Santander	415 (27.3)	81 (28.2)
Valle del Cauca	479 (31.5)	87 (30.3)

Values are presented as number (%). IQR, interquartile range; NA, not applicable.

1 Percentages may not sum to 100 due to rounding.

2 Atlántico included clinics in Barranquilla and Soledad; Santander included clinics in Bucaramanga and Girón; and Valle del Cauca included clinics in Buga, Tuluá, and Palmira.
