# Supplementary material for: Cohort profile: congenital Zika virus infection and child neurodevelopmental outcomes in the ZEN cohort study in Colombia
Source: Epidemiol Health. 2020 Aug 31;42:e2020060. doi: 10.4178/epih.e2020060 (PMC7871158; doi:10.4178/epih.e2020060)
Supplement: Supplementary Material 1. [file epih-42-e2020060-suppl1.docx]

**Supplementary Material 1. Schedule of Study Activities for Pregnant Women, Zika en Embarazadas y Niños (ZEN) Cohort Study (2017-2020)**

| **Trimester of Pregnancy** | **First** | | **Second** | | | | | | | **Third** | | | | | | |  |  |
| --- | --- | --- | --- | --- | --- | --- | --- | --- | --- | --- | --- | --- | --- | --- | --- | --- | --- | --- |
| **Gestational Week^a^** | **10^b^** | **12** | **14** | **16** | **18** | **20** | **22** | **24** | **26** | **28** | **30** | **32** | **34** | **36** | **38** | **Delivery** | **Post-partum visit** | **Sick Visit**^c^ |
| Enrollment Questionnaire | X |  |  |  |  |  |  |  |  |  |  |  |  |  |  |  |  |  |
| Maternal Follow-up Questionnaire |  |  | X |  | X |  | X |  | X |  | X |  | X |  | X |  | X |  |
| Adult Symptoms Questionnaire | X | X | X | X | X | X | X | X | X | X | X | X | X |  | X |  | X | X |
| Ultrasound^d^ | --------X------ | | |  | -----------X--------- | | | |  | ----------------X----------- | | | | |  |  |  |  |
| Venous Blood Sample^e^ | X |  | X |  | X |  | X |  | X |  | X |  | X |  | X | X | X^f^ | X |
| Urine Sample |  | X |  | X |  | X |  | X |  | X |  | X |  |  |  |  |  |  |
| Baseline Venous Blood Sample^g^ | X |  |  |  |  |  |  |  |  |  |  |  |  |  |  |  |  |  |
| Delivery Venous Blood Sample^g^ |  |  |  |  |  |  |  |  |  |  |  |  |  |  |  | X | X^f^ |  |

^a^ This represents an ideal study schedule. Actual visits occurred within a pre-specified window around the ideal visit date that maximized flexibility and reduced participant burden.

^b^ Pregnant women enrolled at any time during the first trimester. The schedule of study activities shifted based on the enrollment date of the pregnant woman. Women were followed every two weeks until the end of pregnancy, alternating between clinic and interval visits.

^c^ Visit occurred if the pregnant woman reported two or more Zika virus compatible symptoms to study or clinic staff outside of study visit schedule.

^d^ If not already conducted, ultrasounds were conducted during the first trimester (at or around the time of the enrollment visit), during the second trimester (between weeks 18-24) and during the third trimester (between weeks 28-36) to monitor fetal health and growth.

^e^ For pregnant women who tested positive for Zika virus by rRT-PCR, women were scheduled to be tested for Zika virus every two weeks until there were two consecutive negative tests, and then they would resume regular study schedule. Women completed the maternal follow-up questionnaire as scheduled and completed a symptoms questionnaire at every blood draw.

^f^ If activities did not occur at delivery, they occurred at the initial postpartum visit.

^g^ Baseline and delivery blood samples were tested for other infections during pregnancy (e.g., syphilis, toxoplasmosis, rubella, cytomegalovirus, herpes, [STORCH]), Zika, dengue, and chikungunya virus by rRT-PCR assay, and Zika or dengue antibodies by IgM assay.
