# Supplementary material for: Cohort profile: congenital Zika virus infection and child neurodevelopmental outcomes in the ZEN cohort study in Colombia
Source: Epidemiol Health. 2020 Aug 31;42:e2020060. doi: 10.4178/epih.e2020060 (PMC7871158; doi:10.4178/epih.e2020060)
Supplement: Supplementary Material 2. [file epih-42-e2020060-suppl2.docx]

**Supplementary Material 2. Schedule of Study Activities for Male Partners, Zika en Embarazadas y Niños (ZEN) Cohort Study (2017-2020)**

| **Partner’s Trimester of Pregnancy** | **First** | | **Second** | | | | | | | **Third** | | | | | | |  |
| --- | --- | --- | --- | --- | --- | --- | --- | --- | --- | --- | --- | --- | --- | --- | --- | --- | --- |
| **Partner’s Gestational Week** | **10^a^** | **12** | **14** | **16** | **18** | **20** | **22** | **24** | **26** | **28** | **30** | **32** | **34** | **36** | **38** | **Delivery** | **Sick Visit** |
| Male Enrollment Questionnaire | X |  |  |  |  |  |  |  |  |  |  |  |  |  |  |  |  |
| Adult Symptoms Questionnaire | X | X |  | X |  | X |  | X |  | X |  |  |  |  |  |  | X |
| Venous Blood Sample | X |  |  |  |  |  |  |  |  |  |  |  |  |  |  |  | X |
| Urine Sample |  | X |  | X |  | X |  | X |  | X |  |  |  |  |  |  |  |
| Semen Sample^b^ |  |  |  |  |  |  |  |  |  |  |  |  |  |  |  |  |  |

^a^ Male partners enrolled in the study up to 1 month after the woman’s enrollment visit, and the schedule of study visits shifted based on the enrollment dates of the pregnant woman and the male partner. Men were followed every month until the end of partner’s second trimester of pregnancy.

^b^ If the male partner tested positive for Zika virus by rRT-PCR, semen samples were collected every 2 weeks until there were two negative ZIKV tests or the end of the partner’s pregnancy.
