# Supplementary material for: Cohort profile: congenital Zika virus infection and child neurodevelopmental outcomes in the ZEN cohort study in Colombia
Source: Epidemiol Health. 2020 Aug 31;42:e2020060. doi: 10.4178/epih.e2020060 (PMC7871158; doi:10.4178/epih.e2020060)
Supplement: Supplementary Material 3. [file epih-42-e2020060-suppl3.docx]

**Supplementary Material 3. Schedule of Study Activities for Infants in 6-Month Follow-up, Zika en Embarazadas y Niños (ZEN) Cohort Study (2017-2020)**

| **Infant Age^a^** | **Birth** | **10 days^b^** | **2**  **weeks** | **4 weeks**  **(1 mo)** | **6**  **weeks** | **8 weeks**  **(2 mo)** | **10 weeks** | **12 weeks**  **(3 mo)** | **14**  **weeks** | **16**  **weeks** | **18**  **weeks** | **20 weeks** | **22**  **weeks** | **24 weeks**  **(6 mo)** | **Sick Visit** |
| --- | --- | --- | --- | --- | --- | --- | --- | --- | --- | --- | --- | --- | --- | --- | --- |
| Infant Symptoms Questionnaire |  | X | X | X | X | X | X | X | X | X | X | X | X | X | X |
| Cranial Ultrasound | X | X^c^ |  |  |  |  |  |  |  |  |  |  |  |  |  |
| Venous Blood Sample | X | X^c^ |  |  |  |  |  |  |  |  |  |  |  |  | X |
| Urine Sample |  |  | X | X | X | X | X | X | X | X | X | X | X | X |  |
| Growth Measurements | X | X | X | X | X | X | X | X | X | X | X | X | X | X |  |
| Routine Eye Exam^d^ |  | X | | | | | | |  |  |  |  |  | X |  |
| RetCam Eye Exam^e^ |  | X | | | | | | |  |  |  |  |  | X |  |
| Hearing Screening |  |  |  | X |  |  |  | X |  |  |  |  |  | X |  |
| Escala Abreviada de Desarrollo, Third Edition |  |  |  |  |  |  |  | X |  |  |  |  |  |  |  |
| Ages and Stages, Third Edition |  |  |  |  |  | X |  |  |  |  |  |  |  | X |  |

^a^ This represents an ideal study schedule. Actual visits occurred within a pre-specified window around the ideal visit date that maximized flexibility and reduced participant burden.

^b^ Initial visit within 10 days of birth.

^c^ If not done at delivery, these samples and tests were obtained at the 10-day visit. If not conducted at the 10-day visit, cranial ultrasound may be conducted up to around 6 months of age.

^d^ Study staff facilitated referrals for all infants to receive an ophthalmologist visit for a standard eye exam within the first three months of age and at 6 months of age.

^e^ All infants with confirmed or possible congenital ZIKV infection, those with an abnormal routine eye exam, and a sample of unexposed infants received RetCam eye exams within the first three months of age and at 6 months of age.
