# Supplementary material for: Cohort profile: congenital Zika virus infection and child neurodevelopmental outcomes in the ZEN cohort study in Colombia
Source: Epidemiol Health. 2020 Aug 31;42:e2020060. doi: 10.4178/epih.e2020060 (PMC7871158; doi:10.4178/epih.e2020060)
Supplement: Supplementary Material 4. [file epih-42-e2020060-suppl4.docx]

**Supplementary Material 4. Schedule of Study Activities for Parents and Children in the 18-Month Follow-up, Zika en Embarazadas y Niños (ZEN) Cohort Study (2017-2020)**

| **Child’s Age^a^** | **6 months** | **9 months** | **12 months**  **(1 year)** | **18 months** |
| --- | --- | --- | --- | --- |
| Parent-Child Enrollment Questionnaire | X |  |  |  |
| Parent-Child Follow-Up Questionnaire |  | X | X | X |
| Growth Measurements (head circumference, height, weight, arm circumference) | X | X | X | X |
| Routine Eye Exam^b^ |  |  | X |  |
| Escala Abreviada de Desarrollo, Third Edition | X | X | X | X |
| Ages and Stages, Third Edition | X^c^ |  | X | X |
| Bayley Scales of Infant & Toddler Development, Third Edition | X | X | X | X |
| Ages and Stages Socio-Economic, Second Edition | X | X | X | X |
| Parenting Stress Index -Short Form -IV | X | X | X | X |
| Centers for Epi Studies Depression Scale 10 | X | X | X | X |

^a^ This represents an ideal study schedule. Actual visits occurred within a pre-specified window around the ideal visit date that maximized flexibility and reduced participant burden.

^b^ Study staff facilitated referrals for all infants to receive a routine eye exam. If child was selected for a RetCam eye exam during the 6-month follow-up but did not receive an exam during the time period, and is enrolled in the 18-month follow-up, then staff facilitated one RetCam exam during the follow-up study visits.

^c^ If not conducted as part of the pregnancy cohort infant developmental screenings (Supplemental Table 1c).
