# Supplementary material for: Cohort profile: congenital Zika virus infection and child neurodevelopmental outcomes in the ZEN cohort study in Colombia
Source: Epidemiol Health. 2020 Aug 31;42:e2020060. doi: 10.4178/epih.e2020060 (PMC7871158; doi:10.4178/epih.e2020060)
Supplement: Supplementary Material 5. [file epih-42-e2020060-suppl5.docx]

**Supplementary Material 5. Characteristics of Pregnant Women who Met Inclusion Criteria, with and without Male Partners, Zika en Embarazadas y Niños (ZEN) Cohort Study (2017-2020)**

|  | | **With Male Partners enrolled**  **N = 287**  **n (%)** | **Without Male Partners enrolled**  **N = 1,232**  **n (%)** | **p-value^a^** |
| --- | --- | --- | --- | --- |
| Gestational age at enrollment | |  |  |  |
| Median gestational week | | 9 (IQR: 7, 12) | 10 (IQR: 8, 12) | 0.4 |
| Age | |  |  |  |
|  | 16-17 | N/A | 120 (10%) | 0.9^b^ |
|  | 18-24 | 135 (47%) | 538 (44%) |  |
|  | 24-34 | 128 (45%) | 482 (39%) |  |
|  | 35+ | 24 (8%) | 92 (7%) |  |
| Highest level of education | |  |  |  |
|  | Primary or less | 41 (14%) | 205 (17%) | 0.01 |
|  | Secondary | 121 (42%) | 609 (49%) |  |
|  | Technical or university | 124 (43%) | 416 (34%) |  |
|  | Missing | 1 (1%) | 2 (0%) |  |
| Type of health insurance | |  |  |  |
|  | Private | 106 (37%) | 343 (28%) | <0.01 |
|  | Public | 174 (61%) | 867 (70%) |  |
|  | Not insured | 3 (1%) | 18 (1%) |  |
|  | Missing | 4 (1%) | 4 (0%^c^) |  |
| Relationship status | |  |  |  |
|  | Married | 55 (19%) | 140 (11%) | <0.01 |
|  | Living together | 227 (79%) | 892 (72%) |  |
|  | Single, divorced, widowed, and other | 5 (2%) | 196 (16%) |  |
|  | Missing | 0 (0%) | 4 (0%) |  |
| Study Site^d^ | |  |  |  |
|  | Atlántico | 119 (41%) | 506 (41%) | 0.87 |
|  | Santander | 81 (28%) | 334 (27%) |  |
|  | Valle del Cauca | 87 (30%) | 392 (32%) |  |

^a^ Pearson chi-squared test for categorical variables or Wilcoxon rank sum test for continuous variables. Missing values were excluded from these calculations.

^b^ Excluded pregnant women <18 years of age from the statistical testing as male partners could not be enrolled if the pregnant woman was <18 years.

^c^ Percentages may not sum to 100 due to rounding.

^d^ Atlántico included clinics in Barranquilla and Soledad; Santander included clinics in Bucaramanga and Girón; and Valle del Cauca included clinics in Buga, Tuluá, and Palmira.
